# Modifying Conventional Psychiatric Practice to Serve Indigenous People

**DOI:** 10.1192/j.eurpsy.2024.1092

**Published:** 2024-08-27

**Authors:** L. Mehl-Madrona, B. Mainguy

**Affiliations:** ^1^Native Studies; ^2^Intermedia, University of Maine, Orono, United States

## Abstract

**Introduction:**

Psychiatry has historically underserved Indigenous people. Earlier, cross-cultural psychiatry assumed that psychiatric disorders were universal and varied little across cultures. We must acknowledge their different views of mind and mental health.

**Objectives:**

In our auto-ethnographic approach, we introduce or re-introduce participants to cultural beliefs, values, and methods for treating addictions, including narrative methods (storytelling), which receive greater acceptance by indigenous and marginalized peoples. Indigenous philosophy states that we see the world using the stories we have absorbed or constructed to explain our perceptions. Using substances is a story that is connected to poverty and adverse childhood events.

**Methods:**

We create new stories to develop a sense of agency, that one’s actions can make a difference in one’s life. We present our experiences and findings from providing psychiatric and addiction services in rural and remote Indigenous settings in Canada (Saskatchewan and Northern Ontario) and in Maine (USA). We present data on a modified approach to psychiatric evaluations and services that emphasizes Indigenous values and begins with a life story interview that determines positive aspects of the client’s history and problem areas and engages the client in therapy from the beginning of the evaluation.

**Results:**

We will demonstrate how this process changes the process of the psychiatric interview, engages Indigenous clients, and results in better outcomes. We discuss how psychotherapy must change to engage Indigenous clients and to be effective with addictions. She will present data on this area. We present the lessons learned and the results of using this approach with a tribal population in Maine. Some key concepts include (1) reframing the person’s self-story about being addicted within a threat-power-meaning network, (2) working with stories about the spirit of the addiction and the consequences of ingesting spirit-laden substances without knowing their songs and protocols, (3) constructing future-self-narratives that explore right relationships and meaningful conduct, (4) constructing stories about the intergenerational transmission of addictions and exploring the question of “whom will be the recipient of your addiction?” We understood that the client sets their goals and defines what recovery means for them, which is the heart of a harm reduction approach.

**Conclusions:**

Indigenous cultures across the world are different but share some similarities including a highly relational approach to defining the self, a collectivist mindset in which the needs of the group can supersede the needs of the individual, a reliance upon stories for transmission of knowledge and culture, and a commitment to a biopsychosocial and spiritual approach, which is often symbolized by the metaphor of the Four Cardinal Directions.

**Disclosure of Interest:**

None Declared

